# Dermoscopy of cutaneous sarcoidosis: a cross-sectional study^☆^

**DOI:** 10.1016/j.abd.2022.12.006

**Published:** 2023-07-22

**Authors:** Mengguo Liu, Huyan Chen, Feng Xu

**Affiliations:** Department of Dermatology, Huashan Hospital, Fudan University, Shanghai, China

**Keywords:** Chronic, Dermoscopy, Granulomatous disease, Sarcoidosis, Skin diseases

## Abstract

**Background:**

Although traditionally used for the diagnosis of skin tumors, in the past few years dermoscopy as a clinical diagnostic aid for inflammatory and infectious skin manifestations has also received more and more attention. The clinical variability of cutaneous sarcoidosis (CS) often makes its correct diagnosis challenging. Dermoscopy can be used as an auxiliary examination method.

**Objective:**

Our aim was to evaluate the role of dermoscopy in the diagnosis and differential diagnosis of CS.

**Methods:**

This was a retrospective analysis of 39 CS clinical and dermoscopic images collected in the Department of Dermatology, Huashan Hospital Affiliated with Fudan University from August 2013 to February 2021.

**Results:**

Retrospective dermoscopic evaluation revealed small grouped, translucent orange globular structures in all 39 cases. Variable diameter linear vessels were found in 38 cases. A central scar-like area was seen in 26 cases. Bright white streaks were seen in 30 cases. The follicular plugs were seen in 15 cases.

**Study limitations:**

First, the number of cutaneous sarcoidosis cases the authors collected is small. Second, due to the lack of a control group, the sensitivity and specificity of the proposed criteria were not calculated. Finally, since our study mainly includes suspicious lesions that were biopsied for diagnostic purposes, there may be a selection bias.

**Conclusion:**

Lesions showing on dermoscopy grouped translucent orange ovoid structures associated with linear vessels should raise the suspicion of CS. Central scar-like areas and bright white streaks are also helpful in the diagnosis of CS.

## Introduction

Sarcoidosis is a multisystemic granulomatous disease with unclear etiology, which can affect a variety of tissues and organs of the body, the most common of which are lungs, lymph nodes, eyes, and skin. The skin manifestations of sarcoidosis are polymorphic, which can be single or multiple, and can be red, yellowish brown, purple spots, papules, plaques, infiltrates, annular lesions, or subcutaneous nodules.^1^ Histopathologically, sarcoidosis is characterized by granuloma of epithelioid histiocytes with few or no inflammatory cells. In addition, epidermal changes, necrosis, foreign bodies, moss infiltration, or granulomatous vasculitis may also occur.

The clinical diversity of Cutaneous Sarcoidosis (CS) brings great difficulties and challenges for dermatologists to correctly diagnose it. In the clinic, many skin diseases can be similar to skin sarcoidosis in appearance, including a series of granulomatous inflammation and infectious skin diseases, such as Lupus Vulgaris (LV), granuloma annular, atypical mycobacterial disease, lipid necrosis, skin leishmaniasis, syphilis, leprosy, foreign body reaction, rheumatoid nodules, and even some tumors from skin accessory sources, such as pilomeduloma, sebaceous adenoma, or trichoepithelioma, and also tumors from the lymphohematopoietic system, such as mycosis fungoides.^2^

Dermatoscope is a non-invasive examination method commonly used in dermatology at present.^3^ It is simple and fast to operate and can clearly show the skin structure invisible to the naked eye of dermatologists. It is called the “stethoscope” of dermatologists. Although it was initially and classically used for the auxiliary diagnosis of skin tumors, in recent years, the application of dermatoscopy in the auxiliary diagnosis of infectious and inflammatory skin diseases has gradually increased. The authors retrospectively analyzed and summarized the dermatoscopic features of 39 patients with CS collected by our department in the past eight years, which may be helpful for clinical diagnosis.

## Methods

This was a retrospective analysis of 39 CS clinical and dermoscopic images collected in the Department of Dermatology, Huashan Hospital Affiliated with Fudan University from August 2013 to February 2021. Dermoscopic images were taken using Dermlite Foto Equipment (3Gen, LLC, Dana Point, Calif., USA) at 10-fold magnification. All the dermoscopic images were acquired before therapy and all the lesions were histopathologically diagnosed by Hematoxylin Eosin (H&E) staining after skin biopsy.

## Results

Thirty-nine patients with 39 lesions of CS were included in this observation (35 women and 4 men; age range = 33∼82 years, mean age = 56.2 years). Thirty-eight cases were type III skin. Fourteen cases occurred on the face. Table 1 summarizes the patients’ characteristics, duration of disease and site of involvement.

**Table 1:** Patient demographics and duration and site of involvement of sarcoidosis

Patient	Sex	Age, years	Clinical diagnosis	Histopathological description	Skin type	Biopsy site
1	F	60	Lupus vulgaris	Cutaneous Sarcoidosis	Ⅲ	Left shoulder
2	F	56	Discoid lupus erythematosus	Cutaneous Sarcoidosis	Ⅲ	Face
3	F	63	Lupus miliaris disseminates faciei	Cutaneous Sarcoidosis	Ⅲ	Face
4	F	59	Cutaneous Sarcoidosis	Cutaneous Sarcoidosis	Ⅲ	Back
5	F	61	Behcet's disease	Cutaneous Sarcoidosis	Ⅲ	Lower limbs
6	F	46	Erythema nodosum	Cutaneous Sarcoidosis	Ⅲ	Lower limbs
7	F	40	Dermatofibrosarcoma protuberans	Cutaneous Sarcoidosis	Ⅲ	Face
8	F	56	Keloid	Cutaneous Sarcoidosis	Ⅲ	Lower limbs
9	F	57	Infectious granuloma	Cutaneous Sarcoidosis	Ⅲ	Face
10	F	56	Cutaneous Sarcoidosis	Cutaneous Sarcoidosis	Ⅲ	Face
11	F	54	Cutaneous tuberculosis	Cutaneous Sarcoidosis	Ⅲ	Face
12	F	60	Unknown	Cutaneous Sarcoidosis	Ⅲ	Face
13	F	44	Keloid	Cutaneous Sarcoidosis	Ⅲ	Left forearm
14	F	53	Discoid lupus erythematosus	Cutaneous Sarcoidosis	Ⅲ	Face
15	F	34	Lupus erythematosus	Cutaneous Sarcoidosis	Ⅱ	Face
16	F	70	Basal cell carcinoma	Cutaneous Sarcoidosis	Ⅲ	Face
17	M	45	Lymphocytic infiltration	Cutaneous Sarcoidosis	Ⅲ	Trunk
18	F	69	Lymphocytic infiltration	Cutaneous Sarcoidosis	Ⅲ	Face
19	F	76	Cutaneous Sarcoidosis	Cutaneous Sarcoidosis	Ⅲ	Face
20	F	52	Mycobacterium infection	Cutaneous Sarcoidosis	Ⅲ	Anterior right ear
21	F	62	Cutaneous Sarcoidosis	Cutaneous Sarcoidosis	Ⅲ	Right neck
22	F	53	Cutaneous Sarcoidosis	Cutaneous Sarcoidosis	Ⅲ	Paranasal
23	F	70	Histiocytosis	Cutaneous Sarcoidosis	Ⅲ	Right upper limb
24	F	68	Cutaneous Sarcoidosis	Cutaneous Sarcoidosis	Ⅲ	Right nasal root
25	F	67	Cutaneous Sarcoidosis	Cutaneous Sarcoidosis	Ⅲ	Forehead
26	F	50	Desmoid tumor	Cutaneous Sarcoidosis	Ⅲ	Left elbow
27	M	55	Folliculitis	Cutaneous Sarcoidosis	Ⅲ	Left anterior chest
28	F	34	Cutaneous Sarcoidosis	Cutaneous Sarcoidosis	Ⅲ	Back
29	F	82	Cutaneous Sarcoidosis	Cutaneous Sarcoidosis	Ⅲ	Face
30	F	49	Cutaneous Sarcoidosis	Cutaneous Sarcoidosis	Ⅲ	Upper limb
31	F	62	Cutaneous Sarcoidosis	Cutaneous Sarcoidosis	Ⅲ	Right cheek
32	F	83	Lymphocytic infiltration	Cutaneous Sarcoidosis	Ⅲ	Right nasolabial sulcus
33	F	51	Nodular vasculitis	Cutaneous Sarcoidosis	Ⅲ	Left upper limb
34	M	33	Angiolymphoid hyperplasia with eosinophilia	Cutaneous Sarcoidosis	Ⅲ	Left upper limb
35	F	55	Cutaneous Sarcoidosis	Cutaneous Sarcoidosis	Ⅲ	Left lower limb
36	M	69	Lupus erythematosus	Cutaneous Sarcoidosis	Ⅲ	Face
37	F	38	Infectious granuloma	Cutaneous Sarcoidosis	Ⅲ	Left eyebrow arch
38	F	48	Peritrichokeratosis	Cutaneous Sarcoidosis	Ⅲ	Left lower limb
39	F	52	Mucinosis	Cutaneous Sarcoidosis	Ⅲ	Back

**Figure 1:**
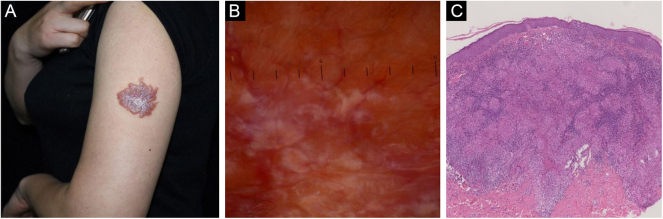
Cutaneous sarcoidosis on the left upper arm. (A) Clinical presentation. (B) Dermoscopic examination reveals diffuse orangish color as well as well-focused linear-irregular vessels; scar-like whitish areas are also visible. (C) Typical histopathological features of sarcoidosis

**Figure 2:**
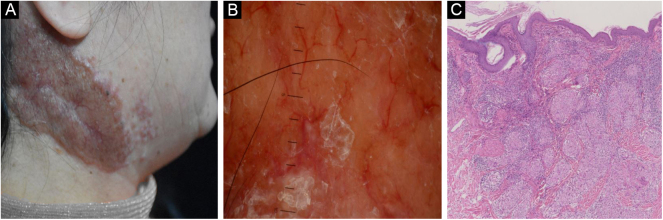
Cutaneous sarcoidosis on the neck. (A) Clinical presentation. (B) Dermoscopic assessment shows focal orangish areas and several well-focused linear/linear-irregular vessels; follicular whitish keratotic plugs and scar-like whitish areas are also present. (C) Typical histopathological features of sarcoidosis

**Figure 3:**
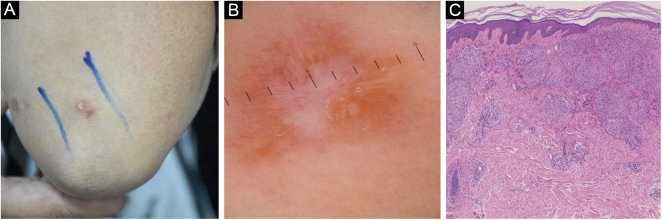
Cutaneous sarcoidosis on the elbow. (A) Clinical presentation. (B) Dermoscopic assessment shows focal orangish, well-focused linear/linear-irregular vessels, whitish linear areas and scar-like whitish areas. (C) Typical histopathological features of sarcoidosis

Retrospective dermoscopic evaluation of the 39 cases of CS revealed small grouped, translucent orange globular structures. Linear vessels of variable diameter were seen in 38 cases (Table 2; Fig. 1). In 26 cases, additional central scar-like areas resembling white structureless areas or white lines in between the translucent orange globules were seen (Figure 2, Figure 3). A follicular plug (Fig. 2) was seen in 15 cases.

**Table 2:** Clinical and dermoscopic features of cutaneous sarcoidosis

Lesion	Clinical color	Grouped translucent orange ovoid structures	Linear vessels	Central scar like areas	Bright white streaks	Follicular plug
Plaque (29/39, 74.4%)	Orange red (7/39, 17.9%)					
Plaque + Ulcer (1/39, 2.6%)	Purplish red (22/39, 56.4%)	39/39, 100%	38/39, 97.4%	26/39, 66.7%	30/39, 76.9%	15/39, 38.5%
Tubercle (9/39, 23.1%)	Dark red (1/39, 2.6%)					
	Pink (9/39, 23.1%)					

Tables Note: The above figures in the table mean: Positive number/total number, percentage.

Linear vessels overlying the translucent orange ovoid structures or within the scar-like areas were the only vascular structures seen in almost all cases of CS (97%). None of the lesions revealed specific dermoscopic criteria of melanocytic skin tumors.

## Discussion

In recent years, more and more studies about the use of dermoscopy in inflammatory and infectious skin diseases have shown its potential role as a diagnostic tool in dermatology. For the skin lesions of various inflammatory and infectious dermatoses, including the diagnosis and differential diagnosis of CS, some relatively characteristic dermoscopy models have been described.^4^, ^5^, ^6^

This study shows that almost all patients of CS (97%) have type III skin, which is different from the European and American populations. At the time of initial diagnosis, only 13 patients were clinically diagnosed as CS. The clinical diagnosis of most patients is not consistent with the final pathological diagnosis. In view of this situation, dermatoscopy can be used as an important consideration for dermatologists in their clinical visits. A dermatoscope can be used as a unique auxiliary diagnostic method for sarcoidosis and can also be used as a tool to suspect this diagnosis.

According to the clinical manifestations of the 39 patients with cutaneous sarcoidosis, most of the lesions were plaques (29/39, 74.4%), a few were nodules (9/39, 23.1%), and only one was plaque and ulcer (1/39, 2.6%). The majority of lesions were purplish red (22/39, 56.4%), a few were pink (9/39, 23.1%), orange-red (7/39, 17.9%), and dark red (1/39, 2.6%). From the perspective of dermatoscopic manifestations, the most common dermatoscopic feature is grouped translucent orange ovoid structures, which are found in all patients (39/39, 100%). The second is linear vessels (38/39, 97.4%), bright white streaks (30/39, 76.9%), and central scarlike areas (26/39, 66.7%). Relatively few are hair follicle angle plugs (15/39, 38.5%). The authors found linear vessels as the only vascular pattern in almost all our cases of CS, and this pattern was always associated with grouped translucent orange ovoid structures, bright white streaks, and central scarlike areas. These findings are consistent with the findings of literature research,^7^, ^8^ but our case number is the largest of all studies that can be consulted at present. Such research results may have important value.

The skin lesions of several skin diseases are very similar to cutaneous sarcoidosis, such as lupus vulgaris, cutaneous leishmaniasis, lipid necrosis and Discoid Lupus Erythematosus (DLE). Their dermatoscopic features also have similarities and differences. One study suggests that dermoscopy can improve the differentiation by revealing predominantly follicular abnormalities in DLE, whereas characteristic orange-yellowish areas/globules and branching arborizing vessels in LV and CS. Although the grouped translucent orange ovoid structures corresponding to the histopathological findings of dermoscopy seems to highly suggest the possibility of cutaneous sarcoidosis, it does not seem to be sufficiently differentiated from other types of granulomatous diseases. CS and LV have similar translucent golden to orange colors, which may be related to the presence of granuloma.

According to a recent study on dermatoscopy of granuloma annulare, the main dermatoscopic clue of this dermatosis is the presence of unfocussed vessels having a variable morphology (dotted in 52.0%, linear-irregular in 44.0%, and/or branching in 28.0% of cases) over a more or less evident pinkish-reddish background.^9^ The most common dermatoscopic findings of cutaneous leishmaniasis include diffuse erythema and vessels, usually having a polymorphic pattern (combination of 2 or more different types of vessels).^10^

## Conclusion

Finally, the authors conclude that the presence of translucent yellow to orange globular-like or structureless areas should raise suspicions of granulomatous dermatosis, especially CS. However, due to the fact that dermatoscopy seems to be insufficient to make a complete diagnosis, established methods such as histopathology, laboratory, or radiological examination are still the status quo of its diagnostic technology.

## Financial support

This work was supported by the 10.13039/501100003399Science and Technology Commission of Shanghai Municipality (nº 18441904300 and 18411952700). Shanghai Municipal Health Commission (nº 2019SY034). Open research funding of Chinese Skin Image Database (nº CSID-ORF-201901 and CSID-ORF-201916). Clinical Research Plan of SHDC (SHDC22022302).

## Authors’ contributions

Mengguo Liu: Performed the statistical analysis, wrote, and revised the manuscript.

Huyan Chen: Took and provided skin pathological pictures.

Feng Xu: Designed the study, and provided the clinical data and images of patients.

## Conflicts of interest

None declared.
